# Effects of Labor Values on Subjective Well-Being: The Mediating Role of Altruistic Tendencies

**DOI:** 10.3389/fpsyg.2021.715179

**Published:** 2021-08-16

**Authors:** Xiaomei Chao, Yuliang Gu

**Affiliations:** ^1^Department of Education, Hunan Normal University, Changsha, China; ^2^Department of Sociology, Hunan Normal University, Changsha, China

**Keywords:** labor values, subjective well-being, altruistic tendency, positive affect, negative affect

## Abstract

This study examined the mediating role of altruistic tendency in the association between labor values and subjective well-being (SWB). About 2,691 Chinese students (1,504 males and 1,187 females) completed the labor values scale (LVS), the Positive Affect and Negative Affect Scale, the Satisfaction With Life Scale, and the altruistic tendency scale. Results demonstrated that labor values were positively associated with life satisfaction and positive affect, while negatively with negative affect. The altruistic tendency was positively correlated with labor values, and positive affect, while negatively correlated with negative affect. Furthermore, altruistic tendency served as a mediator linking labor values and positive/negative affect. These results confirmed the relationship between labor values and SWB and revealed the mechanism of altruism tendency between the two.

Labor values are of great significance in the development of human society. For a long time, the unique value of labor education has been ignored to some extent, and labor education has gradually weakened. As a result, compared with before, young people are unwilling to go to work and cannot work. In response to this situation, the Chinese government issued opinions on comprehensively strengthening labor education in colleges, primary and secondary schools in the new era on March 26, 2020, which emphasizes the importance of labor education. It is proposed that labor education should be included in the whole process of talent training, and integrated with moral education, intellectual education, physical education, and aesthetic education, which will impel students to setup the correct labor values. Why are labor values so important? For primary and middle school students, how will setting up/establishing the positive labor values affect them? What is the specific influence mechanism? Starting from the relationship between labor values and SWB, this study discusses the significance of establishing correct labor values for primary and middle school students. This research specifically studies the influence of labor values on SWB and the mechanism of altruistic tendencies.

## Introduction

### Labor Values and Subjective Well-Being

Values are the ideological system that people use to distinguish good from bad, beauty from ugliness, profit from a loss, right from wrong, and whether they conform to their wishes according to certain standards (Zhang, 2002; Huang and Zheng, 2015). SWB has been conceptualized as the subjective evaluation of work, life, and interpersonal relationships according to his or her subjective standards and feelings, including three dimensions of life satisfaction, positive affect, and negative affect (Diener et al., 2017). Specifically, life satisfaction involves the cognitive evaluation of people of their quality of life, while positive and negative affect involve subjective emotional experience (Diener and Ryan, 2009). Recent studies have shown that values are associated with increased SWB (Rean and Shagalov, 2018). For instance, Yurim et al. (2018) has examined the relationship between values and SWB among nursing students. Results showed that improving the clarity of values could significantly improve individual SWB. A comparative study of SWB in five East Asian regions over the past 20 years has demonstrated that values play an important role in balancing the relationship between income and life satisfaction (Roberts and Clement, 2007; Xie et al., 2017; Lim et al., 2020). As an important prosocial value, labor value determines the value judgment and behavior orientation of an individual to labor (Hao, 2014). Labor values are the general view of the state and degree that labor meets the needs of the people, and it is the internal needs of individuals and the labor characteristics or attributes that they pursue when they engage in activities (Guo and Liu, 2016). The cultivation of labor values is conducive to shaping the good qualities of individuals of being honest and trustworthy, treating others equally, and respecting others in the labor process (Chao and Wang, 2020). Moreover, Duan (2016) has found that labor values have an impact on life satisfaction. In addition, prior research on the transformation of values from “labor glory” to “labor happiness” has confirmed that labor value is also of great significance to the improvement of positive affect (Wang, 2019).

### Altruistic Tendency and SWB

Altruistic behavior, considered the highest level of prosocial behavior, refers to the act of an individual to help others willingly without expecting others to return (Shaw, 1991). It serves as an important variable for SWB development (Pareek and Jain, 2012; Graham, 2014; Zheng and Ya, 2017). Specifically, altruistic behavior leads to more positive social interactions, promotes interpersonal relationships, and strengthens interpersonal trust to enhance the sense of life meaning of individuals and SWB (Stephen, 2005; Grant and Gino, 2010). People with more altruistic behaviors are more likely to be accepted by society and easier to gain support from others, thus obtaining more positive emotional experience, which can be a boost to the SWB of people (Trivers, 1971; Brown, 2003; Piliavin and Sieg, 2007).

The beneficial effects of altruistic behavior on the three dimensions of SWB have been consistently demonstrated by previous studies (Post, 2005; Aknin et al., 2015). For instance, Kahana et al. (2013) have found that altruistic behavior and voluntary service have predicted life satisfaction. Kumar et al. (2012) has demonstrated that active participation in altruistic services can significantly improve the life satisfaction of individuals. The main reason probably is those high altruists (vs. low altruists) are more likely to gain self-affirmation and enhance their sense of self-worth, which leads to higher life satisfaction (Cheng, 2002). In addition, altruistic behavior can also improve the life satisfaction of individuals by providing various forms of social support (Robert et al., 2007). Not only that, but it can also help individuals overcome their inferiority complex and cultivate positive self-worth, thus improving life satisfaction (Adler, 1937).

Besides, altruistic behavior is also positively related to positive affect (Batson, 2010; Akin et al., 2012). Altruistic behavior can improve the SWB of an individual by enhancing the positive affect of an individual (Glomb et al., 2011). Those who prefer to help others and make prosocial behaviors are more inclined to think of themselves as happy people (Lyubomirsky et al., 2005).

While altruistic behavior is considered as a predictor of positive affect, it also contributes to repairing sadness or another negative affect. In support of this idea, Post (2005) has provided empirical evidence that altruistic behavior makes 22% of individuals feel peaceful and effectively alleviates depression. It is precisely because of the important role of altruistic behavior in restoring negative affect that in some psychological treatment, altruistic behavior is considered as an important “helping therapy,” which is especially widely used in the field of social work (Riessman, 1965).

### Labor Values, Altruism Tendency, and SWB

As a psychological trait, individual values have a significant correlation with motivation (Verplanken and Holland, 2002). People first form corresponding behavioral preferences based on values and then produce specific intentions and behaviors under the impact of persistent and stable value preferences (Schwartz, 1992). In other words, positive, friendly values may contribute to guiding prosocial behavior. Specifically, Li and Hou (2013) have found in a sample of young female employees that positive value has a positive predictive effect on altruistic behaviors, that is, those female employees with values of comfort, equality, and innovation tend to engage in more altruistic behaviors. Thus, by inference, as a positive value orientation, labor value can help individuals to possess altruistic tendencies as well. People who respect labor, pay attention to the fruits of labor, and consider labor as an important channel to create happiness are more likely to lend a helping hand to others and make altruistic behavior.

Given that close links between altruistic tendency and SWB, and the significant beneficial effects of altruistic tendencies for SWB (Grant and Gino, 2010; Kahana et al., 2013; Graham, 2014), it can be inferred that labor values may influence the SWB of an individual *via* altruistic tendency.

Although people have built a reasonable relationship between values and SWB, there is no specific discussion on the relationship between labor values and SWB yet, and it is still unclear that how labor values affect SWB. Based on the above discussion, the current study intended to examine the relationship between labor values and SWB and the mediating mechanism of altruistic behavior between them. Furthermore, we, respectively, investigated how labor values would influence the three subdimensions of SWB (i.e., life satisfaction, positive affect, and negative affect) *via* altruistic tendency. Accordingly, we proposed four hypotheses as follows:

H1: Labor values are closely related to SWB. The more positive the labor value is, the higher the SWB level will be.

H1.1 Labor values significantly improved the life satisfaction level of the individuals.

H1.2 Labor values significantly improved the positive affect of the individuals.

H1.3 Labor values significantly restrained the negative affect of the individuals.

H2: Altruistic tendency mediated the influence of labor values on life satisfaction, positive affect, and negative affect.

## Method

### Participants

In the current study, a total of 2,749 students (1,187 females and 1,504 males) were surveyed in two primary schools, two middle schools, and one high school in Dongguan. Except for those who did not complete the questionnaire or had obvious problems in filling out the answers, the effective sample was 2,691 (97.89%). The ages of participants ranged from 9 to 18 (M = 12.50, SD = 2.00). In terms of the grades of students, 48.42% (1,303) of the grades of students were primary school students, 25.01% (673) were junior high school, and 26.57% (715) were senior high school. In terms of the characteristics of the survey object, 1,778 (66.0%) were from rural areas, and 913 (34.0%) were from urban areas. In terms of the social class, under 10 points, 175 (6.5%, score between 1 and 3) were low class, 1,892 (70.3%, score of 4–6 points) were middle class, and 624 (23.2%, scores between 7 and 10 points) were high class.

### Measures

#### Labor Values

Labor values were assessed by the labor values scale (LVS; Chao and Wang, 2020), which was a 15-item self-report scale based on five aspects (e.g., honest labor value, equality status of labor value, cherishing labor value, loving labor value, and distribution value according to work). Sample items were “I enjoy the labor process” and “I have equal respect for workers of different occupations, whether they are cleaners or engineers, etc.” The responses of students were measured on a 5-point Likert-type scale that ranged from 1 (not at all) to 5 (very much). Higher scores indicate a more positive labor value. Since the scale was prepared based on the higher grade of primary school, confirmatory factor analysis was used in this study to explore the applicability of this scale in middle school and high school groups. The confirmatory factor analysis indexes were as follows: $\chi_{\left(78\right)}^{2}$ = 7.118, *P* <0.001, GFI = 0.93, comparative fit index (CFI) = 0.92, root-mean-square error of approximation (RMSEA) = 0.076, standardized root-mean-square residual (SRMR) = 0.0583. All the indexes were in line with the requirements of surveying. The Cronbach's α of the whole scale was 0.832.

#### Positive Affect and Negative Affect

SPANE was used to assess two dimensions of SWB such as positive and negative affect (Karim et al., 2011). The SPANE is a 1 (very slightly/not at all) to 5 (extremely) 5-point scale, consisting of 5 items measuring positive affect (e.g., excited) and 10 for negative affect (e.g., nervous, sad). This scale has demonstrated good reliability and validity in China (Li and Hou, 2013; Xiang et al., 2020). In the present study, the Cronbach's α for the positive affect and negative affect subscales were 0.841 and 0.863, respectively.

#### Life Satisfaction

Life satisfaction was assessed by the Satisfaction With Life Scale (SWLS; Diener et al., 1985). The SWLS is a 7-point Likert scale ranging from 1 = strongly disagree to 7 = strongly agree. For each participant, higher scores indicated higher levels of life satisfaction. For example, items are “I am satisfied with my life,” “If I could go back in time, I wouldn't change anything.” The Chinese version of the scale has been demonstrated to have high reliability and validity in the Chinese population (Kong et al., 2012; Zhao et al., 2014). In this study, the Cronbach's α of the scale was 0.824.

#### Altruistic Tendency

The altruistic tendency was assessed by using items related to altruistic behavior factors in the Organizational Citizenship Behavior scale (OCB; Coyle-Shapiro, 2002). The scale has five items ranging from 1 = strongly disagree to 5= strongly agree (e.g., “help the students who ask for leave to finish the task they should have finished”). A total summed score of all the items was calculated and higher scores indicated higher levels of altruistic tendency. In the present study, Cronbach's α was 0.795.

## Results

### Measurement Model

The measurement model consisted of five latent variables (labor value, altruistic tendency, life satisfaction, and positive and negative affect) and 13 observational variables. The analysis results of the measurement model indicated that the fitting data index of the model reaches the standard [χ^2^_(55, 2, 691)_ = 347.543, *p* < 0.001; RMSEA = 0.044; SRMR = 0.0285; CFI = 0.977]. Table 1 showed the descriptive statistical results and correlation analysis results. The results showed that the relationship between labor values and altruistic tendency (*r* = 0.045, *p* < 0.05), life satisfaction (*r* = 0.426, *p* < 0.01), positive affect (*r* = 0.056, *p* < 0.01), and negative affect (*r* = −0.052, *p* < 0.01) could be significantly correlated. Altruistic tendency and life satisfaction (*r* = 0.045, *p* < 0.05), positive affect (*r* = 0.373, *p* < 0.01) and negative affect (*r* = −0.512, *p* < 0.01) were significantly correlated; Positive affect and life satisfaction (*r* = 0.073, *p* <0.01) and negative affect (*r* = −0.512, *p* < 0.01) were significantly correlated.

**Table 1:** Descriptive statistics and zero-order correlations for all variables (*N* = 2691).

	***M* ±*SD***	**1**	**2**	**3**	**4**	**5**
Labor Value	59.03 ± 8.65	1.000				
Altruistic Tendency	21.24 ± 5.30	0.045^*^	1.000			
Life Satisfaction	23.13 ± 6.95	0.426^***^	0.045^*^	1.000		
Positive Affect	17.65 ± 4.22	0.056^**^	0.373^***^	0.073^**^	1.000	
Negative Affect	10.45 ± 4.15	−0.052^**^	−0.224^***^	−0.024	−0.512^***^	1.000

* *p < 0.05,*

** *p < 0.01,*

*** *p < 0.001*.

### Structural Model

**Figure 1:**
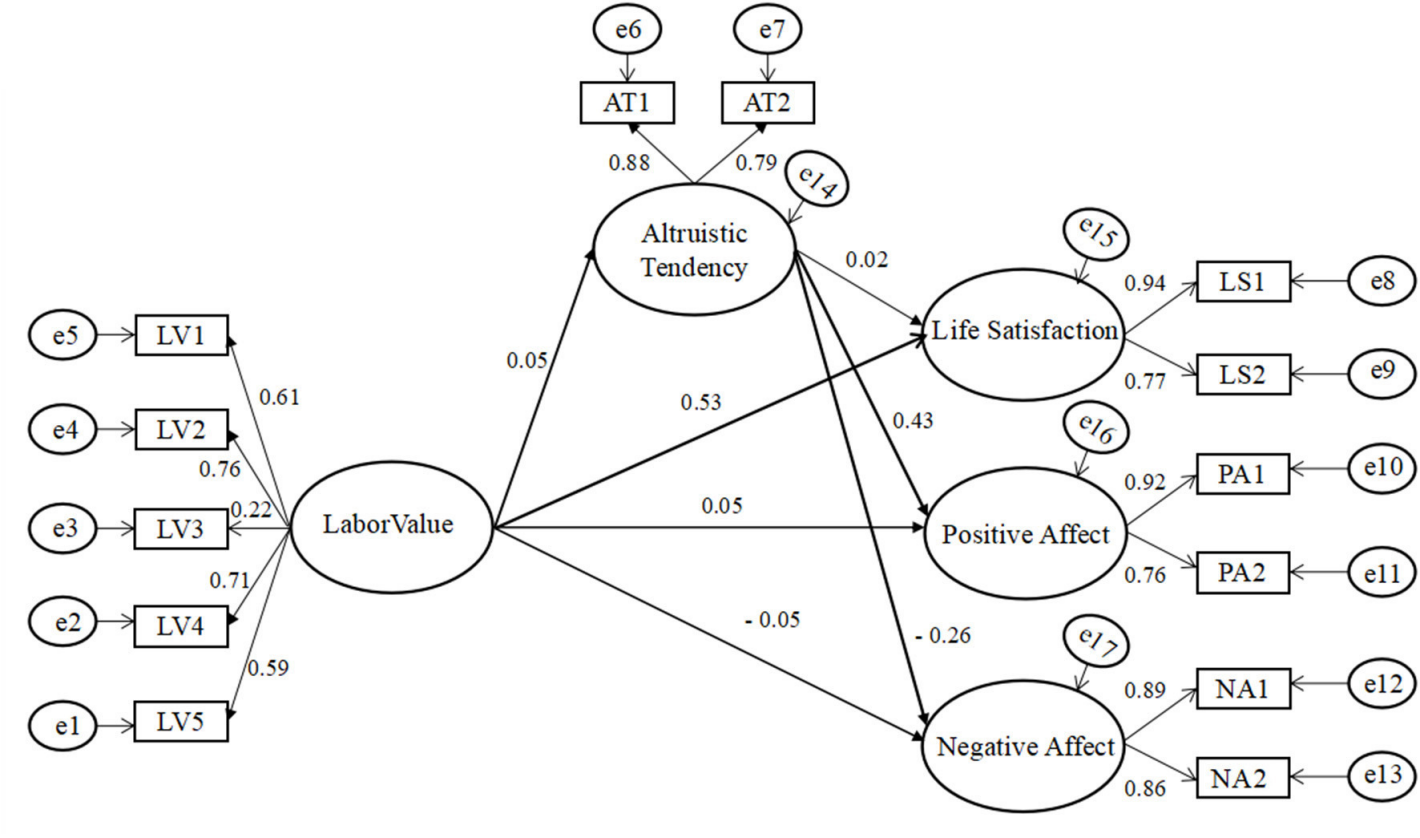
The factor loadings in the mediation model are standardized.

Labor values significantly and directly predicted life satisfaction (β = 0.426, *p* < 0.001), positive affect (β = 0.056, *p* < 0.01), and negative affect (β = −0.052, *p* < 0.01), when mediating variable altruistic tendency was not present. Then we established a structural model (Model 1) based on the hypothesis. In this model, labor values directly predicted the scores of life satisfaction, positive and negative affect, and indirectly predicted the scores of all three through altruistic tendency (as shown in Figure 1). The results showed that the fitting degree of Model 1 was good [χ^2^_(55, 2, 691)_ = 347.543, *p* < 0.001; RMSEA = 0.044; SRMR = 0.0285; CFI = 0.977] (see Table 2). Therefore, we used Model 1 as the structural model.

**Table 2:** Fit indices of Model 1.

	**χ^**2**^**	***df***	**CFI**	**RMSEA**	**SRMR**	**AIC**	**ECVI**
Model 1	347.543	55	0.977	0.044	0.0285	419.543	0.156

*CFI, comparative fit index; RMSEA, root mean square error of approximation; SRMR, standardized root mean square residual; AIC, Akaike information criteria; ECVI, expected cross-validation index*.

### Test of the Mediation Model

At the same time, we further used the bootstrap method to, respectively, test the mediating effect of altruistic tendency between labor value, life satisfaction, and positive and negative affect in Model 1. About 1,000 bootstrap samples were extracted from the original data set (*N* = 2.691). The results indicated that altruistic tendency played a significant and independent mediating role in the association between labor values and positive affect [95% CI = (0.002, 0.069)] and also played a significant and independent mediating role in the association between labor values and negative affect [95% CI = (−0.039, −0.001)] (as shown in Table 3).

**Table 3:** Standardized indirect effects and 95% confidence intervals.

**Pathways**	**Estimate**	**Lower**	**Upper**
Labor Value → Altruistic Tendency → Life Satisfaction	0.002	−0.001	0.010
Labor Value → Altruistic Tendency → Positive Affect	0.034	0.002	0.069
Labor Value → Altruistic Tendency → Negative Affect	−0.018	−0.039	−0.001

### Sex Differences

To test the stability of the structural model, the cross-sex stability of the model is further analyzed. First, we tested whether there were sex differences in the five potential variables. The results showed that altruism tendency [*t*_(2, 691)_ = 0.475, *p* > 0.05], life satisfaction [*t*_(2, 691)_ = 0.213, *p* > 0.05], positive affect [*t*_(2, 691)_ = −0.254, *p* > 0.05], and negative affect [*t*_(2, 691)_ = 0.578, *p* > 0.05] had no significant sex difference while labor value [t _(2, 691)_ = −3.135, *p* < 0.05] had. Male scores on labor value are lower than female scores.

Moreover, based on sex differences that have been found, multigroup confirmatory factor analysis was used to explore the stability of the model. In this study, based on keeping the basic parameter factor load, error variance, and structural covariance unchanged, we established two models, one of which allowed free estimation of paths across sexes (unconstrained structural path), and the other restricted equality of path coefficients between the two sexes (constrained structural path). Results showed that there was no significant difference between the two models [χ^2^_(7, 2, 691)_ = 4.961, *p* = 0.665]. At the same time, the fitting indexes of the two models all reached the fitness standard (as shown in Table 4). In addition, to avoid that χ^2^ reached a significant level because of its susceptibility to a large sample size, critical ratios of differences (CRD) were used as an indicator to further investigate the cross-sex stability of the structural model. According to the decision rules, if the absolute value of CRD is >1.96, the two parameters are significantly different (Arbuckle, 2003). The results showed that there was no significant difference in the structural paths of all the variables, which explained there was no significant difference in the cross-sex comparison between the two models, that is, the model has cross-sex stability.

**Table 4:** Comparison of constrained and unconstrained structural path models for transgender.

	**χ^**2**^**	***df***	**CFI**	**RMSEA**	**SRMR**	**AIC**	**ECVI**
Unconstrained structural path	457.247	139	0.975	0.029	0.0315	543.247	0.202
Constrained structural path	462.208	146	0.975	0.028	0.0316	534.208	0.199

*CFI, comparative fit index; RMSEA, root mean square error of approximation; SRMR, standardized root mean square residual; AIC, Akaike information criterion; ECVI, expected cross-validation index*.

## Discussion

The present study used the questionnaire method to examine the relationships among labor values, altruistic tendencies, and SWB. Results found that labor values were positively correlated with life satisfaction and positive affect while negatively correlated with negative affect. The altruistic tendency was positively correlated with labor values, life satisfaction, and positive affect while was negatively correlated with negative affect. Furthermore, the altruistic tendency played a mediating role in the relationship between labor values and positive/negative affect while did not mediate the relations between labor values and life satisfaction.

First, we examined the association between labor values and SWB. Consistent with hypothesis 1, labor values were positively correlated with SWB. Meanwhile, labor values were also closely related to positive emotion, negative emotion, and life satisfaction, which are three dimensions of SWB. Results were consistent with previous research showing that positive values positively predicted the life satisfaction of individuals (Ma and Ding, 2020). Positive values assist people in maintaining an optimistic attitude and gaining more positive feedback from others, causing the improvement of positive affect (Lim et al., 2020). Previous studies have demonstrated that positive values can effectively alleviate individual anxiety and depression (Luo and Cheng, 2015; Yang et al., 2019). Deci and Ryan (2000) has found that positive values were negatively correlated with individual psychological distress index, that is, positive values can significantly inhibit psychological distress. In addition, Chao and Wang (2020) have more directly found that positive labor values cut down the risk of individuals falling into negative affect.

Additionally, the findings also revealed that altruistic tendency served as an important mediator between labor values and SWB, which was in line with hypothesis 2. Specific to the three dimensions of SWB, altruistic tendency played a significant mediating role between labor values and positive/negative affect. Individual behavior and its values are consistent across time and environment (Vecchione et al., 2016; Ponizovskiy et al., 2019). Those individuals with positive labor values show more excellent qualities in life, such as kindness, friendliness, and honesty, and often lead to positive behaviors consistent with these excellent qualities (Li and Hou, 2013). The positive labor values are helpful to guide the emergence of altruistic tendencies, which is helpful to improve the SWB of individuals. High altruists are more likely to be accepted by society and get support from others, thus obtaining more positive emotional experiences, which is conducive to the improvement of SWB. The dual experimental analysis from internal and external pathways has shown that altruistic tendency can not only enhance the positive affect of individuals but also effectively inhibit the negative affect of individuals. From the internal perspective of the individual, the altruistic tendency is a self-motivation process, which promotes the positive interaction between the body and mind of the individual. That is, the helpers get their positive biological feedback because of the implementation of altruistic tendencies to enhance the positive feelings (Hu et al., 2016; Li and Xie, 2017). In addition, the altruistic tendency can also help individuals reduce or even eliminate the negative affect such as panic, sadness, anxiety, and inferiority complex, as well as the negative interpersonal cognitive bias caused by these negative emotions through the mechanism of empathy (Pan et al., 2013; Shen, 2014; Xiao et al., 2014). From the external perspective of the individual, the altruistic tendency can show the excellent personal qualities (e.g., trustworthiness and dependence) of the helper to others, so that the helper can obtain positive feedback in social communication, reciprocal cooperation, mate selection, and other aspects (Barclay, 2010; Bereczkei et al., 2010; Fehrler and Przepiorka, 2013; Moore et al., 2013). In turn, this positive feedback further eliminates individual negative emotional experiences and improves the self-efficacy of helpers (Mogilner et al., 2012).

Interestingly, we did not find that altruistic tendency plays a mediating role between labor values and life satisfaction. The reason may be that the overly strong predictive relationship of labor values to life satisfaction (β = 0.53) inhibits the mediation mechanism of altruistic tendency. Of course, it may also be caused by the limitations of the samples collected in this research, and the possible reasons need to be discussed further.

We confirmed the relationship between labor values and SWB and discussed the mediating role of altruism in the relationship between labor values and SWB. The research has contributed to revealing the influence of labor values on SWB and its specific mechanism. At the same time, the research also responds to the importance of implementing labor education for primary and middle school students and training them to establish correct labor values. Primary and secondary school students are forming their values and world views through school education. Understanding the importance of setting up correct labor values will make it more necessary and legitimate to carry out labor values education for primary and middle school students. However, there are also some limitations to the study. Due to the limitation of conditions, this study is a cross-sectional study, and whether the research conclusions can be extended to other age groups of students remains to be further tested. In addition, the research objects are primary and secondary school students in China. Due to the differences in national systems and cultural backgrounds, further studies are needed to demonstrate whether the research conclusions can be used to explain the relationship between labor values and SWB of primary and secondary school students in other countries and cultural systems. Future studies can further examine the relationship between the variables through systematic validation and longitudinal follow-up of subjects of different ages. The scope of interpretation of research conclusions can also be discussed through cross-cultural comparative studies. The data used in this study are all from the subjective reports of the subjects, so there may be errors due to the interference of various situations and social factors. Future research can consider various ways to collect data to improve the objectivity of measurement.

## Data Availability Statement

The original contributions presented in the study are included in the article/supplementary files, further inquiries can be directed to the corresponding author/s.

## Ethics Statement

The studies involving human participants were reviewed and approved by Ethics Committee of Hunan Normal University. Written informed consent to participate in this study was provided by the participants' legal guardian/next of kin. Written informed consent was obtained from the minor(s)' legal guardian/next of kin for the publication of any potentially identifiable images or data included in this article.

## Author Contributions

All authors listed have made a substantial, direct and intellectual contribution to the work, and approved it for publication.

## Conflict of Interest

The authors declare that the research was conducted in the absence of any commercial or financial relationships that could be construed as a potential conflict of interest.

## Publisher's Note

All claims expressed in this article are solely those of the authors and do not necessarily represent those of their affiliated organizations, or those of the publisher, the editors and the reviewers. Any product that may be evaluated in this article, or claim that may be made by its manufacturer, is not guaranteed or endorsed by the publisher.
